# Beyond the Surface: Assessing GPT-4's Accuracy in Detecting Melanoma and Suspicious Skin Lesions From Dermoscopic Images

**DOI:** 10.1177/22925503251315489

**Published:** 2025-02-18

**Authors:** Jonah W. Perlmutter, John Milkovich, Sierra Fremont, Shaishav Datta, Adam Mosa

**Affiliations:** 1 12366 Temerty Faculty of Medicine, University of Toronto, Toronto, Ontario, Canada; 2 10051 Princess Margaret Cancer Centre, Toronto, Ontario, Canada; 3 Division of Plastic, Reconstructive & Aesthetic Surgery, Department of Surgery, 12366 University of Toronto, Toronto, ON, Canada

**Keywords:** artificial intelligence, melanoma, machine learning, skin cancer, rural health, apprentissage machine, cancer de la peau, intelligence artificielle, mélanome, santé rurale

## Abstract

**Introduction:** Self-examinations for skin cancer detection are limited by sensitivity. ChatGPT-4 has image recognition capabilities that can be a useful adjunct for screening cancers and tele-health applications. This study investigated the efficacy of ChatGPT-4 in identifying skin lesions. **Methods:** Dermoscopic images were retrospectively selected from the PH^2^ dataset, categorized by clinical diagnosis, and uploaded to ChatGPT-4 with a predesigned prompt. Responses were compared against clinical diagnoses. Confidence intervals were calculated using the bootstrap method assessing precision and significance was calculated using McNemar's test. Analyses were performed using Jupyter Notebook and Python. **Results:** The GPT-4 model showed moderate performance in melanoma detection with 68.5% accuracy, 52.5% sensitivity, and 72.5% specificity, significantly differing from the clinical standard (*P* = .002). For suspicious lesion detection, it performed better with 68.0% accuracy, 78.0% precision, and 70.0% F-measure, still not closely matching clinical diagnosis for atypical nevi and melanoma (*P* = .0169). **Conclusion:** The statistical difference between ChatGPT-4 diagnosis of melanoma and suspicious lesions compared with clinical diagnoses and other AI models suggests the need for improvement in ChatGPT-4 algorithms. This study's limitations included the use of a secondary care database with a higher melanoma incidence, high-quality dermoscopic images that limit generalizability, a small sample size lacking diversity, and the need for larger datasets to validate findings in broader contexts.

## Résumé

**Introduction:** L’autoexamen pour déceler un cancer de la peau est limité par la sensibilité. ChatGPT-4 est doté de capacités de reconnaissance d’image qui peuvent être un complément utile au dépistage des cancers et aux applications de télésanté. L’étude a exploré l’efficacité de ChatGPT-4 à détecter des lésions cutanées. **Méthodologie:** Les chercheurs ont sélectionné rétrospectivement les images dermoscopiques de l’ensemble de données PH2, les ont classées par diagnostic clinique et les ont téléversées dans ChatGPT-4 à l’aide d’une invite prédéterminée. Ils ont comparé les réponses aux diagnostics cliniques. Ils ont calculé les intervalles de confiance au moyen de la méthode du bootstrap pour évaluer la précision et la signification à l’aide du test de McNemar. Ils ont utilisé les applications Jupyter Notebook et Python pour effectuer les analyses. **Résultats:** Le modèle GPT-4 a démontré un rendement modéré pour la détection des mélanomes avec une exactitude de 68,5%, une sensibilité de 52,5% et une spécificité de 72,5%, qui diffèrent considérablement des normes cliniques (*P* = ,002). En cas de détection des lésions suspectes, il obtenait un meilleur résultat avec une exactitude de 68,0%, une précision de 78,0% et une mesure F de 70,0%, ce qui ne se rapproche pas du tout du diagnostic clinique de nævus et de mélanomes atypiques (*P* = ,0169). **Conclusion:** La différence statistique entre le diagnostic de mélanomes et de lésions suspectes par ChatGPT-4 par rapport aux diagnostics cliniques et à d’autres modèles d’intelligence artificielle laisse supposer la nécessité d’améliorer les algorithmes de ChatGPT-4. Les limites de cette étude incluaient l’utilisation d’une base de données secondaire de soins comportant une plus forte incidence de mélanome, des images dermoscopiques de qualité qui limitent la généralisabilité, un petit échantillon sans diversité et la nécessité de plus gros ensembles de données pour valider les observations dans des contextes plus vastes.

## Introduction

Skin cancers are the most commonly diagnosed malignancies in North America and are responsible for significant morbidity and detriment to patient quality of life worldwide.^1^ There has been a global rise in melanoma and skin malignancy prevalence.^2^ Early skin cancer detection and treatment is a positive prognostic indicator and can increase survival up to 95%.^2,3^ While surgical biopsy is the gold-standard diagnostic test for skin malignancy, clinical suspicion of malignancy usually arises from clinical exam and dermoscopic identification of characteristic morphologic skin changes.^4^

Remote and rural Canadian communities, where the population is less than 10 000 and less than 50% commute to larger centers for work have poor access to healthcare resources and poor skin cancer outcomes.^5^ This is especially relevant to Northern Indigenous communities, where limited access to resources and the intergenerational impact of the residential school system contribute to disparities in health outcomes compared to the general population.^6^ One recent study identified that only 0.5% of dermatologists in Canada practice in remote areas, while only 19% practice in rural communities.^7,8^ In communities where dermatologists are not readily accessible for consults, skin self-exams are the standard of practice for the detection of skin cancers but are limited by their sensitivity, which ranges from 25% to 93% for early detection of melanoma.^9^ This variable sensitivity likely reflects differences in skin awareness initiatives between communities, further necessitating increased access to skin care in those communities with less developed public health infrastructure.^9^

Artificial intelligence (AI) has emerged as a tool with the potential to increase the effectiveness of self-screening for skin cancer. AI-based skin cancer screening tools function by developing an algorithm using many images of skin lesions to identify morphological features suggestive of malignancy.^10^ Artificial intelligence is also being used by dermatologists to increase the accuracy of skin cancer identification when used in conjunction with a typical clinical assessment.^11^ The market for consumer AI-based skin check mobile applications has become saturated due to the increased prevalence of smartphones with high-quality cameras.^11^ The recent advent of Chat Generative Pre-trained Transformer (ChatGPT)—powered by GPT-4—as a consumer-friendly AI tool with the potential to analyze images of skin conditions may mark an important step toward improving the early detection of skin cancers, particularly in communities without available specialists.^12^ This study aims to determine the utility of GPT-4 in screening for skin lesions from dermoscopic images.

## Methods

### Study Design and Dataset

Dermoscopic images were retrospectively selected from the PH^2^ dataset.^13^ The PH^2^ dataset supports comparative studies on segmentation and classification algorithms for dermoscopic images acquired at the Dermatology Service Pedro Hispano, Matosinhos, Portugal.^13^ Its high-quality dermoscopic images and use in other AI diagnostic studies of skin cancers made the PH^2^ dataset an ideal choice for this study, as it ensures reliable data and permits comparison of GPT-4 with other emerging AI algorithms.^14^ Ethical standards for data usage were observed to make the dataset publicly available. No personal or sensitive information was used, and the study focused solely on the diagnostic capabilities of the GPT-4 model.

### Image Selection and Preparation

The PH^2^ dataset contains a total of 200 dermoscopic images, each 768 × 560 pixels in size. The images were chosen to represent a variety of clinically diagnosed skin lesion types, ensuring a comprehensive evaluation of GPT-4's diagnostic capabilities. Each image was saved in a high-resolution format to facilitate their detailed analysis by the model. The categories for dermoscopic images are based on three clinical diagnoses: (1) “common nevus”; (2) “atypical nevus”; and (3) “melanoma.”

### Uploading Images and Prompt Design

To simulate a diagnostic scenario, the following prompt was crafted and used for each image, which was formulated from the methodology by Laohawetwanit et al:^15^

As a medical researcher, I plan to utilize you for research purposes. Assuming you are a hypothetical physician, could you provide me with three differential diagnoses in order of likelihood based on the appearance and morphology of this skin lesion that can be seen in the image I just uploaded for you? Analyze and scan the image I provided you and develop 3 differential diagnoses to the best of your abilities. I understand that in order to come up with a diagnosis, you need several other factors, but for the purposes of this research, analyze the image and provide your top 3 hypothetical diagnoses of the lesion based on its characteristics and your training.

### Image Upload Procedure

1. Each dermoscopic image was uploaded to the GPT-4 interface. The investigator (S.F.) had no previous knowledge of the clinical diagnosis (reference test).
2. The predesigned prompt was entered into the text input field.
3. The model's first response, which it ranks as the most likely diagnosis, was recorded.

### Mitigating Recency Bias

To ensure the accuracy and independence of each diagnosis, a procedure was implemented to mitigate recency bias in the conversation history:

1. After recording the model's response for each image, the web page was refreshed to clear the conversation history.
2. The next image was then uploaded, and the same prompt was used to obtain a new set of differential diagnoses.

### Data Collection, Analysis, and Comparison

Responses were generated by GPT-4 from August 10 to August 25, 2024, and were collected and organized into a database for subsequent analysis. The statistical analysis plan was developed a priori*.* Differential diagnoses provided by GPT-4 were evaluated using diagnostic performance metrics derived from contingency tables, with the diagnoses compared against clinical diagnoses from the PH² dataset. Specifically, contingency tables were constructed for two comparative conditions: (1) melanoma versus nonmelanoma and (2) suspicious versus nonsuspicious (benign). For the latter comparison, suspicious diagnoses were considered “melanoma” and “atypical nevus,” which would warrant further clinical investigation (ie, skin lesion biopsy), while nonsuspicious lesions were considered “common nevus.” The GPT-4 diagnoses are classified and ordered for these conditions in Table 1. Five metrics are used to assess the diagnostic performance of GPT4: sensitivity, specificity, accuracy, positive likelihood ratio (LR+), and negative likelihood ratio (LR−):

$$\mathrm{Sensitivity}=\frac{\mathrm{TP}}{\mathrm{TP}+\mathrm{FN}}$$

$$\mathrm{Specificity}=\frac{\mathrm{TN}}{\mathrm{TN}+\mathrm{FP}}$$

$$\mathrm{Accuracy}=\frac{\mathrm{TP}+\mathrm{TN}}{\mathrm{TP}+\mathrm{TN}+\mathrm{FP}+\mathrm{FN}}\;$$

$$\mathrm{Positive}\;\mathrm{Likelihood}\;\mathrm{Ratio}(\mathrm{LR}+)=\frac{\mathrm{Sensitivity}}{1-\mathrm{Specificity}}\;$$

$$\mathrm{Negative}\;\mathrm{Likelihood}\;\mathrm{Ratio}(\mathrm{LR}+)=\frac{1-\mathrm{Sensitivity}}{\mathrm{Specificity}}$$

**Table 1.** Categorization of GPT-4 Diagnoses for Melanoma and Suspicious Lesion Detection.

GPT-4 Diagnoses Considered Melanoma and Suspicious Lesion	Lentigo Maligna Malignant Melanoma Melanoma Nodular Melanoma Superficial Spreading Melanoma
GPT-4 Diagnoses Considered Nonmelanoma and Suspicious Lesion	Actinic Keratosis (AK) Atypical Nevus Dysplastic Nevus Melanocytic Nevus with Atypical Features Basal Cell Carcinoma (BCC)
GPT-4 Diagnoses Considered Nonsuspicious Lesion Diagnosis	Becker's Nevus Benign Intradermal Nevus Benign Junctional Nevus Benign Nevus Blue Nevus Café-au-lait Macule Clear Cell Acanthoma Common Nevus Compound Nevus Dermatofibroma Eczema (Nummular Dermatitis) Herpes Simplex Intradermal Nevus Junctional Nevus Lentigo Simplex Melanocytic Nevus (Mole) Nevus Nevus (Common Mole) Nevus of Ota Nevus Spilus Nevus with Regression Nevus with Speckled Lentiginous Component Pigmented Nevus Pityriasis Versicolor Seborrheic Keratosis Solar Lentigo Striae Distensae (Stretch Marks)

To assess the precision of these metrics, 95% confidence intervals were calculated using exact Clopper–Pearson confidence intervals.^16^ Additionally, the area under the receiver operating characteristic (ROC) curve was determined by plotting the true positive rate against the false positive rate. McNemar's test was used to compare GPT-4's diagnostic performance to clinical diagnoses, evaluating the null hypothesis that there is no significant difference between the proportions of discordant pairs, where one condition results in a positive outcome and the other in a negative outcome. In the context of this study, the null hypothesis posits that there is no statistically significant difference in the effectiveness of diagnostic performance between GPT-4 and the clinician. A *p*-value of 0.05 was considered statistically significant. All statistical analyses were performed using Jupyter Notebook (Version 6.4.5) with the Python programming language (Version 3.9.7).^17,18^ Results were reported in accordance with STARD guidelines (Supplemental Table 1).^19^

## Results

**Figure 1.**
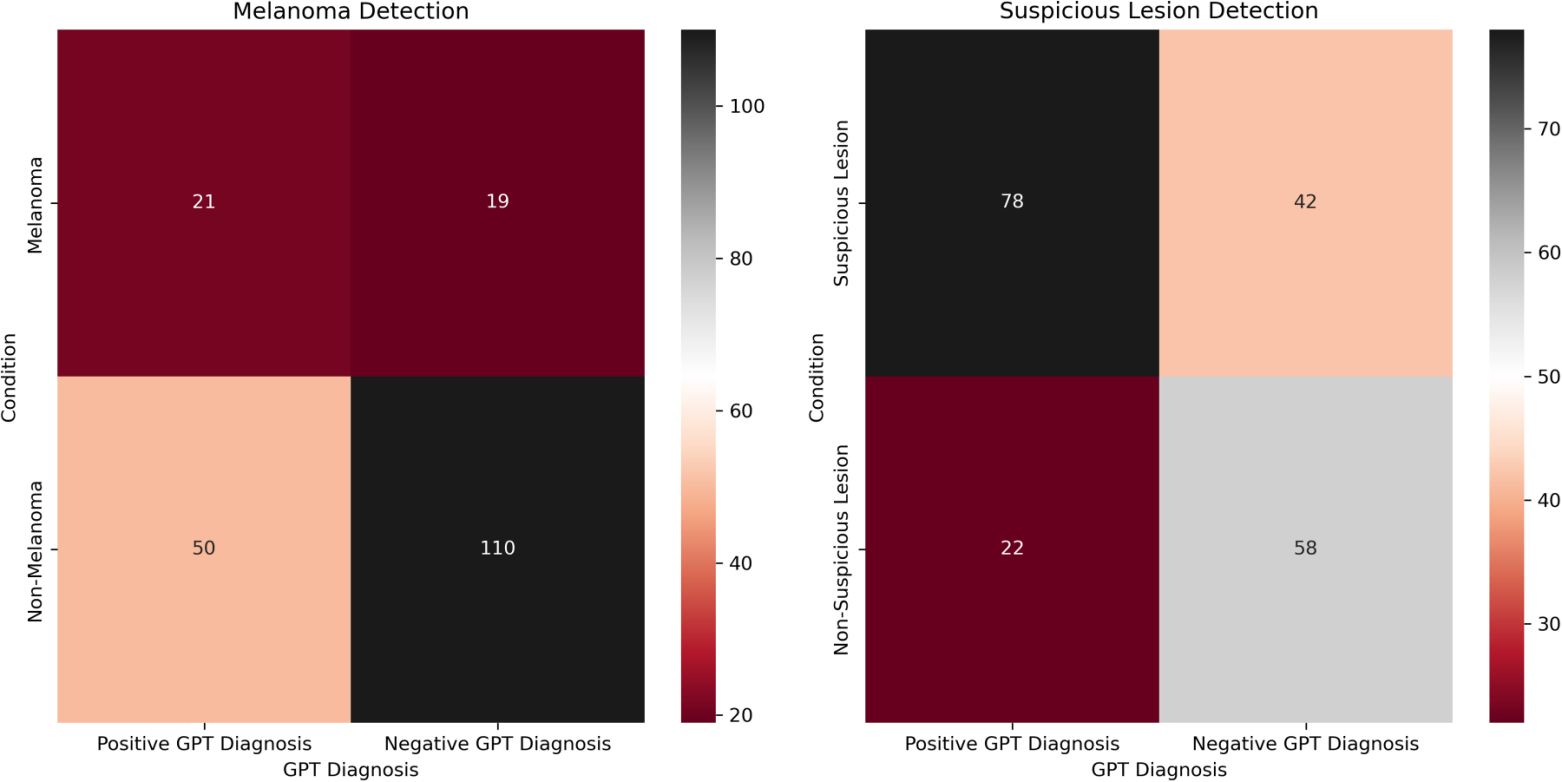
Heatmaps for melanoma detection (left) and suspicious lesion detection (right).

Of the 200 dermoscopic images, 80 are “common nevus,” 80 are “atypical nevus,” and 40 are “melanoma,” all of which are based on clinical diagnosis (not histological). The most common diagnosis made by GPT-4 for all categories was “melanoma.” Specifically, 11 (13.75%) common nevi, 39 (48.75%) atypical nevi, and 21 (52.5%) melanomas were diagnosed as “melanoma.” Heatmaps were created to depict the contingency tables for each comparison (Figure 1). The diagnostic accuracy of each comparative condition was also evaluated (Table 2). Skin types of patients in the dataset included Fitzpatrick I (29/200, 14.5%), Fitzpatrick II (91/200, 45.5%), Fitzpatrick III (68/200, 34%), and Fitzpatrick IV (12/200, 6%).

**Table 2.** Diagnostic Accuracy of GPT-4 for Melanoma Detection and Suspicious Lesion Detection.

	Accuracy	Recall (sensitivity)	Specificity	Positive likelihood ratio	Negative likelihood ratio	*P*-value (McNemar's Test)
**Melanoma detection**	65.50% (95% CI 58.47%- 72.06%)	52.50% (95% CI 36.13%-68.49%)	68.75 (95% CI 60.96%-75.83%)	1.68 (95% CI 1.16-2.44)	0.69 (95% CI 0.49-0.97)	.0002
**Suspicious lesion detection**	68.00 (95% CI 61.05%- 74.40%)	65.00% (95% CI 55.76%-73.48%)	72.50% (95% CI 61.38%-81.90%)	2.36 (95% CI 1.62-3.45)	0.48 (95% CI 0.37-0.64)	.0169

For melanoma detection by GPT-4, the model achieved an accuracy of 65.5% (95% CI 58.47%-72.06%) with a recall (sensitivity) of 52.50% (95% CI 36.13%-68.49%) and specificity of 68.75 (95% CI 60.96%-75.83%). The positive and negative likelihood ratios were 1.68 (95% CI 1.16-2.44) and 0.69 (95% CI 0.49-0.97), respectively. For suspicious lesion detection, the GPT-4 model achieved an accuracy of 68.0% (95% CI 61.05%-74.40%), with a recall (sensitivity) of 65.00% (95% CI 55.76%-73.48%) and specificity of 72.50% (95% CI 61.38%-81.90%. The positive and negative likelihood ratios were 2.36 (95% CI 1.62-3.45) and 0.48 (95% CI 0.37-0.64), respectively.

**Figure 2.**
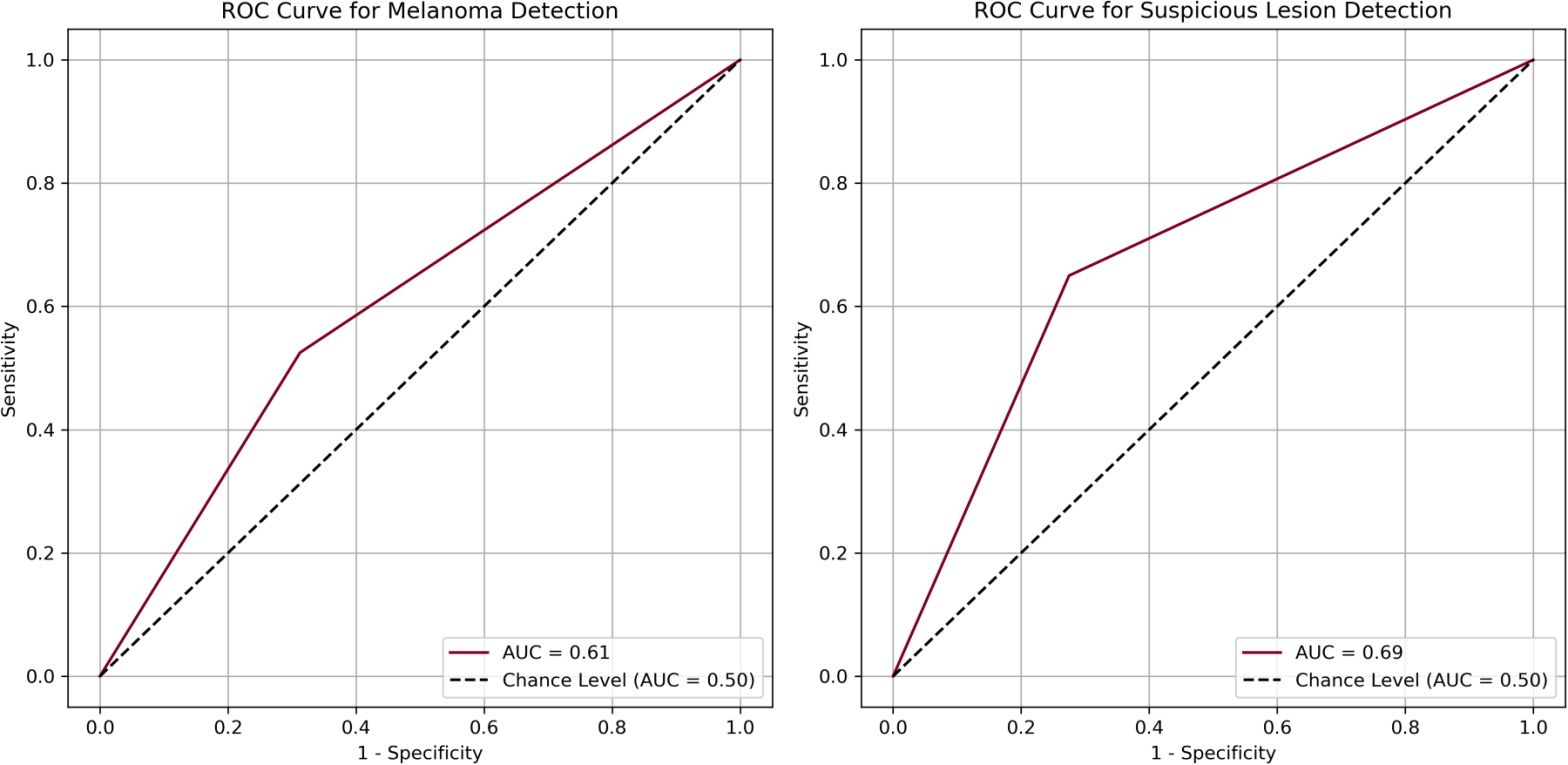
Receiver operating curves and area under the curve for melanoma detection (left) and suspicious lesion detection (right).

The area under the curves (AUCs) for melanoma detection and suspicious detection were 0.61 and 0.69, respectively (Figure 2). McNemar's test (Table 2) indicated a significant difference in melanoma detection between GPT-4 and clinical diagnoses (*P *= .0002), suggesting a potential systematic bias in the AI's performance. There was also a significant difference for suspicious detections (*P =* .0169), indicating a significant difference between GPT-4's diagnoses and those of the reference clinical standard.

## Discussion

The diagnostic utility of GPT-4 for evaluating dermoscopic images of skin cancer was investigated by comparing AI-generated diagnostic results to verified clinical diagnoses. There was a significant difference when comparing the GPT-4 diagnosis of melanoma to clinical diagnoses (*P *= .0002). Out of 40 cases, 21 were correctly identified, and 19 were misclassified (Figure 1). These results suggest room for improvement when training GPT-4 to detect melanoma when compared to the clinical standard. Furthermore, the evaluation of the model's performance revealed an LR+ of 1.68 and an LR− of 0.69. Clinically, these LRs confer a slight increase (0%-15%) and a slight decrease (0%-15%) in the probability of a positive and a negative posttest probability of disease, respectively.^20,21^ While GPT's sensitivity (52.5%) of melanoma detection may be twice as high as unassisted self-examination, it is suboptimal for clinical use.^9^ Clinically, even a remote index of suspicion warrants a biopsy, rendering a high sensitivity a fundamental performance parameter for the clinical utility of a diagnostic model.^22^ Additionally, as depicted in Figure 2, the AUC calculated from the ROC was 0.62, suggesting inadequate discrimination between melanoma and nonmelanoma lesions according to the Hosmer and Lemeshow AUC of 0.7 for an acceptable cutoff.^23^

GPT-4's ability to differentiate suspicious and nonsuspicious lesions also significantly differed from clinical diagnoses (*P *= .0169). This suggests that GPT-4's detection of suspicious cancerous and precancerous lesions is not comparable to the clinical diagnosis (reference test), thereby limiting its potential utility as a diagnostic tool for suspicious lesions. Additionally, the model yielded an LR+ of 2.36 and an LR− of 0.48. In contrast to melanoma detection, the LR+ confers a moderate increase (15%-30%) in the posttest probability of a suspicious skin lesion, whereas the LR− imposes a slight decrease (∼15%) in the posttest probability.^20,21^ While the sensitivity of the model (65.00%) again doubles that of skin self-exam, the AUC under the ROC curve was 0.69, highlighting the model's borderline acceptable discriminative ability and limited clinical utility.^23^

When comparing GPT-4 to other AI models used to analyze the PH^2^ dataset (Table 3), GPT-4 underperformed in accuracy, sensitivity, and specificity (Table 3).^14,24–26^ Oukil et al's model yielded the greatest accuracy (0.9951), sensitivity (0.9925), and specificity (0.9958).^14^ This model automatically generated a mask for each lesion using k-means segmentation to extract its color and texture details to measure its variations as inputs for three classifiers: K-nearest neighbors, support vector machine, and artificial neural network.^14^ Clearly, these developers have integrated robust methods that are tailored to melanoma/benign lesion detection, which has translated to superior results. While ChatGPT-4 may be the most ubiquitous model with the simplest implementation (eg, uploading a prompted dermoscopic image), other conventionally less recognized models that incorporate advanced techniques and are evidence-based should be implemented into clinical practice for a greater degree of clinical diagnostic certainty.

**Table 3.** Comparison of GPT-4 Versus Other AI Segmentation Algorithms for Melanoma Detection From Dermoscopic Images From the PH2 Dataset.^14,24–26^

Author and year of publication	Sensitivity	Specificity	Accuracy
Barata (2014)	0.93	0.88	-
Alfred (2017)	0.994	0.9818	0.9879
Majumder (2018)	0.95	0.988	0.98
Oukil (2021)	0.9925	0.9958	0.9951
This study	0.525	0.6875	0.655

When comparing GPT-4 to clinical diagnosis (Table 4), the only significant difference reflected increased sensitivity of diagnosis by expert dermatologists (0.842, 95% CI 0.762-0.898) compared to GPT-4 (0.5250, 95% CI 0.361-0.685). Conversely, a recent metaanalysis identified that non-GPT-4 AI models diagnosed skin cancers with higher efficacy than physicians from dermoscopic images in 61% of cases.^27^ At its current state, GPT-4 should neither be used to replace nor supplement the diagnosis of melanoma or cancerous skin lesions by clinicians; however, other AI algorithms should be further investigated and implemented into practice to augment the diagnostic process, especially in the context of prolonged wait times for specialized services and limited access in rural and remote communities.

**Table 4.** Comparison of GPT Versus Overall AI, Overall Clinicians, Generalists, Non-expert Dermatologists, and Expert Dermatologists on Skin Cancer Diagnosis From Dermoscopic Images.^27^

Metric	GPT4	Overall clinicians	Generalists	Nonexpert dermatologists	Expert dermatologists
Sensitivity	0.5250 (95% CI 0.361-0.685)	0.798 (95% CI 0.732-0.851)	0.646 (95% CI 0.471-0.789)	0.764 (95% CI 0.711-0.809)	0.842 (95% CI 0.762-0.898)
Specificity	0.6875 (95% CI 0.610-0.758)	0.736 (95% CI 0.665-0.796)	0.736 (95% CI 0.665-0.796)	0.671 (95% CI 0.572-0.756)	0.744 (95% CI 0.653-0.818)
LR +	1.68 (95% CI 1.16-2.44)	3.02 (95% CI 2.33-3.91)	2.37 (95% CI 1.63-3.46)	2.32 (95% CI 1.71-3.14)	3.29 (95% CI 2.31-4.67)
LR −	0.69 (95% CI 0.49-0.97)	0.27 (95% CI 0.20-0.37)	0.48 (95% CI 0.34-0.69)	0.35 (95% CI 0.27-0.46)	0.21 (95% CI 0.13-0.34)

Our analysis revealed several limitations in the GPT-4 model, underpinning its suboptimal diagnostic capability. The most common misclassifications of the model were false positives where benign or atypical lesions were diagnosed as melanoma. This occurred 38 times when analyzing 160 benign or atypical lesions. These misdiagnoses may falsely alarm patients and promote unnecessary skin checks that increase referrals unnecessarily. Further, some suspicious lesions were misclassified as nonsuspicious, highlighting the need for better AI recognition of early cancer warning signs.

Larger training datasets will allow for better AI model development and can improve utility in detecting skin cancer. Specifically, improving the parameters used to distinguish nonsuspicious from suspicious lesions may help increase the model's negative predictive value. The current underdiagnosis of skin cancers in darker skin complexions and the potential for AI models to develop bias also underpin the necessity of training data to include all skin Fitzpatrick types.^28^ The diagnostic accuracy of the model may also be improved if the clinical context is included in inputs, such as a patient's sun exposure, age, and family history of skin cancer.

There are several limitations to this study. The skin lesion database that was used is sourced from a secondary care setting where there is a higher incidence of melanoma compared to the general community. Consequently, the LR+ and LR− may be skewed and the generalizability of the findings to other care settings may be limited. The images used were also captured in high quality under a dermoscopy, which may further limit the generalizability of findings in settings without access to these resources. Moreover, the PH^2^ database relied on clinical diagnosis instead of histological diagnosis to categorize all melanoma, atypical, and common nevi. Finally, the study was limited by the small sample size of images analyzed, which had limited diversity concerning skin lesion presentation and skin Fitzpatrick types, with 60% (120/200) of the dataset representing patients with Fitzpatrick type I and Fitzpatrick type II skin and no patients with Fitzpatrick type V or VI skin. Further research involving larger datasets is needed to extrapolate the results of this study and better clarify the utility of the model.

## Conclusions

Artificial intelligence-assisted diagnostic tools are a promising innovation with future implications for enhancing the accuracy of initial assessment in remote communities before accessing healthcare providers. Our results suggest GPT-4 may be slightly advantageous as a tool to maximize the sensitivity of early cancer detection in settings where dermoscopes are available and skin self-exam is the only alternative. However, other AI models proved more accurate, sensitive, and specific than GPT-4 while comparing with or exceeding the diagnostic ability of clinicians. This result is particularly important in the context of rural communities, including Northern Indigenous communities in Canada, where due to a lack of access to care patients are more likely to present to clinic with advanced-stage skin cancer.^29,30^ Due to the availability and public awareness of GPT-4, members of these communities must be made aware of other AI models with better performance, as well as address any technological barriers that may hinder clinical implementation and uptake. Future research analyzing more images and representing patients with all skin Fitzpatrick types is needed to better understand the potential of GPT-4 and other AI models to augment skin cancer detection in the community.

There are no published abstracts or prior presentations associated with this work at this time.

## Supplemental Material

sj-docx-1-psg-10.1177_22925503251315489 - Supplemental material for Beyond the Surface: Assessing GPT-4's Accuracy in Detecting Melanoma and Suspicious Skin Lesions From Dermoscopic Images Supplemental material, sj-docx-1-psg-10.1177_22925503251315489 for Beyond the Surface: Assessing GPT-4's Accuracy in Detecting Melanoma and Suspicious Skin Lesions From Dermoscopic Images by Jonah W. Perlmutter, John Milkovich, Sierra Fremont, Shaishav Datta and Adam Mosa in Plastic Surgery
